# Cerebral Blood Flow Alterations in Type 2 Diabetes Mellitus: A Systematic Review and Meta-Analysis of Arterial Spin Labeling Studies

**DOI:** 10.3389/fnagi.2022.847218

**Published:** 2022-02-16

**Authors:** Jieke Liu, Xi Yang, Yong Li, Hao Xu, Jing Ren, Peng Zhou

**Affiliations:** Department of Radiology, Sichuan Cancer Center, School of Medicine, Sichuan Cancer Hospital and Institute, University of Electronic Science and Technology of China, Chengdu, China

**Keywords:** type 2 diabetes mellitus, arterial spin labeling, cerebral blood flow, meta-analysis, seed-based *d* mapping

## Abstract

**Objective:**

Arterial spin labeling (ASL) studies have revealed inconsistent regional cerebral blood flow (CBF) alterations in patients with type 2 diabetes mellitus (T2DM). The aim of this systematic review and meta-analysis was to identify concordant regional CBF alterations in T2DM.

**Methods:**

A systematic review was conducted to the published literatures comparing cerebral perfusion between patients with T2DM and healthy controls using ASL. The seed-based *d* mapping (SDM) was further used to perform quantitative meta-analysis on voxel-based literatures and to estimate the regional CBF alterations in patients with T2DM. Metaregression was performed to explore the associations between clinical characteristics and cerebral perfusion alterations.

**Results:**

A total of 13 studies with 14 reports were included in the systematic review and 7 studies with 7 reports were included in the quantitative meta-analysis. The qualitative review found widespread CBF reduction in cerebral lobes in T2DM. The meta-analysis found increased regional CBF in right supplementary motor area and decreased regional CBF in bilateral middle occipital gyrus, left caudate nucleus, right superior parietal gyrus, and left calcarine fissure/surrounding cortex in T2DM.

**Conclusion:**

The patterns of cerebral perfusion alterations, characterized by the decreased CBF in occipital and parietal lobes, might be the neuropathology of visual impairment and cognitive aging in T2DM.

## Introduction

Type 2 diabetes mellitus (T2DM) is a common metabolic disease in middle-aged and older adults characterized by chronic hyperglycemia, which leads to long-term macrovascular and microvascular complications of various organ systems. The epidemic of T2DM and its complications raise a global health threat (Zheng et al., 2018). The present literatures have proved that T2DM is a significant risk factor of developing certain mental disorders, including cognitive dysfunction, dementia, and depression (Biessels and Despa, 2018; van Sloten and Schram, 2018; Xue et al., 2019), and older individuals with T2DM progress to dementia at faster rates (Xu et al., 2010; Morris et al., 2014). Although the underlying mechanisms of these disorders are still unaddressed, growing evidences indicate that cerebral microvascular dysfunction is one of the key mechanisms, which may be driven by hyperglycemia, obesity, insulin resistance, and hypertension (van Sloten et al., 2020). Therefore, characterizing the phenotype of cerebral perfusion alterations may advance our understanding of the underlying mechanisms of cognitive aging and mental impairments in T2DM.

As the brain is a highly metabolic organ with limited energy reserves, the metabolically active regions need abundant supply of glucose and oxygen *via* cerebral perfusion (Coucha et al., 2018). Cerebral blood flow (CBF), commonly defined as the volume of blood delivered to a unit of brain tissue per minute, is responsible for the delivery of nutrients to the brain (Fantini et al., 2016). CBF is also correlated to brain activity, and there is a coupling between metabolically active regions and CBF under normal circumstances (Hoge et al., 1999). Recent studies have observed neurovascular decoupling in T2DM (Hu et al., 2019; Yu et al., 2019; Zhang et al., 2021). Therefore, the cerebral perfusion impairment may cause oxidative metabolism dysfunction of brain and neuronal damage, leading to mental disorders in T2DM.

CBF can be quantitatively measured using single-photon emission computerized tomography (SPECT), positron emission tomography (PET), perfusion computed tomography (PCT), dynamic susceptibility contrast magnetic resonance imaging (DSC-MRI), and arterial spin labeling (ASL). However, SPECT and PET require injection of radiotracers while PCT and DSC-MRI require injection of intravenous contrast agent (Wintermark et al., 2005). Besides, SPECT, PET, and PCT are associated with radiation exposure. Compared with the aforementioned methods, ASL is a non-invasive method to measure CBF by magnetically labeling the inflowing arterial blood water *in vivo* as an endogenous tracer (Williams et al., 1992). Due to its non-radiation, non-invasiveness, and reliability, ASL is proposed as a promising method to reveal cerebral perfusion biomarkers in various mental disorders (Alsop et al., 2015; Haller et al., 2016; Zhang, 2016).

In the last three decades, growing literatures have attempted to characterize cerebral perfusion patterns in T2DM, but the findings are varied across studies. A recent study systematically reviewed literatures on cerebral perfusion in T2DM and found the reduction of regional cerebral perfusion in multiple locations, including occipital lobe, domains involved in the default mode network and the cerebellum (Wang et al., 2021). However, this study involved various modalities including SPECT, DSC-MRI, and ASL. More importantly, no quantitative synthesizing method was used to conduct meta-analysis of voxel-based studies. As the region of interest (ROI) method has inherent bias and is more liberal in statistical threshold than voxel-based analysis (VBA) method (Radua and Mataix-Cols, 2009; Yao et al., 2021), the quantitative meta-analysis of voxel-based studies can objectively identify regional CBF differences at whole-brain level without any *priori* hypothesis.

Therefore, we first systematically reviewed literatures on cerebral perfusion in T2DM using ASL and then conducted a quantitative meta-analysis on these voxel-based literatures using Seed-based *d* Mapping (SDM, formerly Signed Differential Mapping) as primary tool. The SDM is a well-recognized synthesizing method for voxel-based studies and has been used in meta-analysis of cerebral structural and functional alterations in T2DM (Liu J. et al., 2017; Liu et al., 2021; Yao et al., 2021). This study aimed to identify consistent regional CBF alterations in T2DM and explore the potential effects of the clinical characteristics on these perfusion alterations.

## Methods

### Search Strategy and Study Selection

A systematic search was conducted for relevant studies in the PubMed, Web of Knowledge, and Embase databases before November 30, 2021 according to the Preferred Reporting Items for Systematic reviews and Meta-Analyses (PRISMA) guidelines (Page et al., 2021a,b). The keywords were (“diabetes” or “diabetic”) and (“arterial spin labeling” or “ASL”). Besides, the references of the retrieved studies and suitable reviews were manually checked for additional eligible studies.

Studies were included in systematic review according to the following criteria: (1) Original article published in peer-reviewed journal and in English; (2) conducted group comparison between patients with T2DM and healthy controls; (3) measured whole-brain or regional CBF using ASL. Studies were further included in meta-analysis according to the additional criteria: (1) Used VBA to estimate CBF changes; (2) reported coordinates of significant clusters in Montreal Neurological Institute (MNI) or Talairach space. The exclusion criteria were as follows: (1) studies that re-analyzed previously published data; (2) studies without available full-text record; (3) studies that only reported ROI findings or without available coordinates were further excluded in meta-analysis.

For each included study in systematic review, the extracted information included sample size, gender, age, comorbidity, brain regions and their CBF alterations. For each included study in meta-analysis, additional information was recorded as follows: (1) Clinical characteristics including years of education, diabetic duration, onset age, body mass index (BMI), hemoglobin A_1c_ (HbA_1c_), and Mini Mental State Examination (MMSE) score; (2) acquisition parameters including scanner, sequence, labeling duration, post labeling delay (PLD), and spatial resolution; (3) analytic methods including software package, full width at half maximum (FWHM), partial volume effect (PVE) correction, and statistical threshold. The corresponding author were contacted *via* email for additional data that were required in the meta-analysis. Two radiologist (JL and XY) independently conducted the literature search and extracted data. The discrepancies between the two radiologists were resolved by consensus.

### Voxel-Based Meta-Analysis

Voxel-based meta-analysis was conducted with SDM software package (version 5.15)^1^. The procedures including the data preparation, preprocessing, mean analysis, and statistic test were summarized here in brief (Radua and Mataix-Cols, 2009; Radua et al., 2012, 2014).

First, the peak coordinates and *t*-values were written in a text file for each study. Only the peak coordinates at the whole-brain level were extracted to avoid biases toward liberally thresholded brain regions in ROI studies (Friston et al., 2006; Radua and Mataix-Cols, 2009). The studies with non-statistically significant unreported effects (NSUEs) were also included, and their text files were recorded with no content and named with the extension of “.no_peaks.txt.” Second, an anisotropic non-normalized Gaussian kernel was used to recreate an effect-size map and its variance map for each study. Both positive and negative coordinates were reconstructed in the same map to avoid any voxel erroneously appearing positive and negative simultaneously. The FWHM was set at 20 mm as it was found to optimally balance the sensitivity and specificity in SDM, according to previous simulations (Radua et al., 2012). Third, the mean map was obtained by performing a voxel-wise calculation of the mean of the study maps, weighted by the sample size, the inverse of the variance of each study, and the inter-study heterogeneity. Finally, the statistic test was conducted with the default SDM threshold, which were proposed to optimally balance sensitivity and specificity and to be an approximate equivalent to a corrected *P*-value of 0.05 for effect-size in SDM (*p* < 0.005, peak height *z* = 1, cluster extent > 50 voxels) (Radua and Mataix-Cols, 2009; Radua et al., 2012).

### Reliability, Heterogeneity and Publication Bias Analyses

The jackknife sensitivity analysis was performed to test the replicability of the results by iteratively repeating the analyses, discarding one dataset each time. We presumed that the findings might be highly conclusive and replicable if previous significant results could be replicated in all or most study combinations.

The inter-study heterogeneity of each significant cluster was tested using a random-effects model. Magnitude of heterogeneity was estimated using *I*^2^ index, computed as 100% × (*Q*—*df*)/*Q*, where *df* is the degree of freedom, which estimated the proportion of variability due to non-random differences between studies. The value of *I*^2^ less than 25% indicated low heterogeneity (Higgins et al., 2003).

The funnel plot of each significant cluster was created by Egger’s test to estimate the publication bias. The result with *p* < 0.05 was considered significant for publication bias (Egger et al., 1997).

### Subgroup Meta-Analysis

To explore the potential biases that were introduced by the different acquisition parameters and analytic methods between the studies, we conducted subgroup analyses. We repeated the analysis for those studies acquiring images with pulsed ASL (PASL), with pseudo-continuous ASL (PCASL), and with a slice thickness 4 mm. We also repeated the analysis for those studies using PVE correction.

### Metaregression Meta-Analysis

The potential effects of relevant clinical variables on regional brain CBF alterations in patients with T2DM were examined by a random-effects general linear metaregression. The independent variables explored by the metaregression included percentage of males, mean age, years of education, diabetic duration, onset age, body mass index (BMI), hemoglobin A_1c_ (HbA_1c_), Mini Mental State Examination (MMSE) score. The dependent variable was the SDM-Z value. As reported in a previous study, we decreased the probability threshold to 0.0005 to reduce false positives (Radua and Mataix-Cols, 2009). In the findings of metaregression analysis, the regions that did not overlap with those in the main between-group analysis were discarded. Finally, regression plots were visually inspected to discard fittings driven by few studies (Radua and Mataix-Cols, 2009; Radua et al., 2012).

## Results

### Included Studies and Sample Characteristics

**FIGURE 1:**
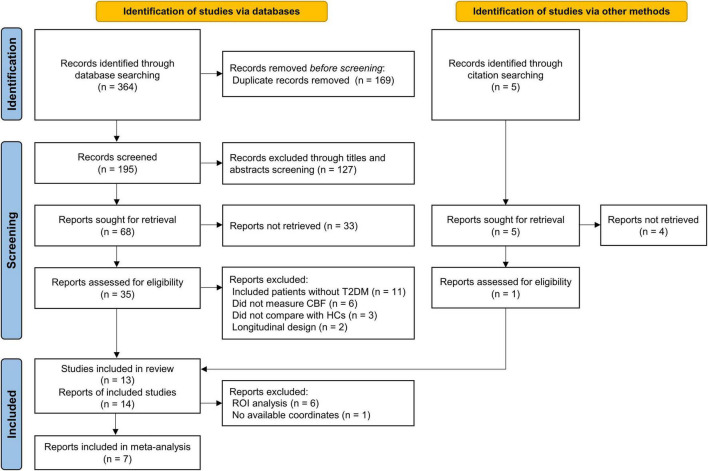
PRISMA flow diagram for literature search and study selection. PRISMA, Preferred Reporting Items for Systematic reviews and Meta-Analyses; T2DM, type 2 diabetes mellitus; HCs, healthy controls; CBF, cerebral blood flow; ROI, region of interest.

A total of 369 records were identified through database searching and citation searching, and Figure 1 shows the flowchart of literature search and study selection. We finally included 13 studies with 14 reports in the systematic review (Last et al., 2007; Jor’dan et al., 2014; Novak et al., 2014; Rusinek et al., 2015; Xia et al., 2015; Jansen et al., 2016; Cui et al., 2017; Dai et al., 2017; Shen et al., 2017; Bangen et al., 2018; Zhang et al., 2019; Chau et al., 2020; Huang et al., 2021). One study performed analysis using both ROI and VBA methods (Jansen et al., 2016). As 1 of 8 VBA reports had no available coordinate (Novak et al., 2014), 7 studies with 7 reports were finally included in the meta-analysis (Xia et al., 2015; Jansen et al., 2016; Cui et al., 2017; Dai et al., 2017; Shen et al., 2017; Zhang et al., 2019; Huang et al., 2021).

The search revealed 407 patients with T2DM and 443 healthy controls in the systematic review and 253 patients with T2DM and 247 healthy controls in the meta-analysis. The basic characteristics of the studies in the systematic review including sample size, gender, age, comorbidity, and the main findings of brain regions and their CBF alterations are summarized in Table 1. The relevant clinical characteristics, acquisition parameters, and analytic methods of the included studies in the meta-analysis are presented in Tables 2, 3.

**TABLE 1:** Arterial spin labeling studies investigating cerebral blood flow alterations in patients with T2DM relative to healthy controls.

References	T2DM			Healthy controls		Method	Brain regions	CBF alteration
	No. (male/female)	Age (years)	Comorbidity (No.)	No. (male/female)	Age (years)			
Last et al. (2007)	26 (13/13)	61.6 ± 6.6	Hyperlipidemia (10), hypertension (10), retinopathy (10)	25 (13/12)	60.4 ± 8.6	ROI	Frontal, temporal, and parieto-occipital lobe	↓
Jor’dan et al. (2014)	61 (31/30)	65 ± 8	Hyperlipidemia (34), hypertension (38), peripheral neuropathy (31)	67 (28/39)	67 ± 9	ROI	Cerebellum, frontal, temporal, parietal, and occipital lobe	n.s.
Novak et al. (2014)	15 (8/7)	62.0 ± 7.9	Hyperlipidemia (10)	14 (4/10)	60.1 ± 9.9	VBA	Insular cortex	↓
Rusinek et al. (2015)	23 (9/14)	54.2 ± 5.2	NA	37 (15/22)	51.8 ± 3.8	ROI	Frontal and parietal lobe	n.s.
Xia et al. (2015)	38 (17/21)	56.0 ± 6.1	Hypertension (29)	40 (21/19)	57.1 ± 7.6	VBA	R middle occipital gyrus, R and L inferior parietal lobe, R precuneus	↓
Jansen et al. (2016)	41 (NA)	NA	Hypertension (39), cardiovascular disease (8)	39 (NA)	NA	ROI	Whole cerebral cortex, frontal, temporal, parietal, and occipital cortex, and subcortical gray matter	n.s.
						VBA		n.s.
Cui et al. (2017)	40 (21/19)	60.5 ± 6.9	Lacunar infarcts (9)	41 (13/28)	57.9 ± 6.5	VBA	Dorsal anterior cingulate cortex	↑
							R and L middle occipital gyrus, R precuneus, cuneus	↓
Dai et al. (2017)	41 (19/22)	65.5 ± 8.3	Hypertension (32)	32 (16/16)	67.3 ± 10.1	VBA	Cerebellum, frontal lobe	↓
Shen et al. (2017)	36 (17/19)	57.6 ± 6.2	Hyperlipidemia (9), hypertension (20), white matter hyperintensities (29)	36 (14/22)	56.2 ± 6.8	VBA		n.s.
Bangen et al. (2018)	11 (8/3)	72.3 ± 2.8	Hypertension (11), cardiovascular disease (1), atrial fibrillation (1)	38 (13/25)	73.6 ± 5.9	ROI	R and L hippocampus, R inferior parietal cortex, R inferior temporal cortex, R rostral middle frontal gyrus	↓
							L inferior parietal cortex, L inferior temporal cortex, R and L medial orbitofrontal cortex, L rostral middle frontal gyrus	n.s.
Zhang et al. (2019)	26 (10/16)	51.9 ± 10.7	Hyperlipidemia (9), hypertension (7), cardiovascular disease (1)	26 (11/15)	48.2 ± 6.7	VBA	R temporopolar, R superior and middle frontal gyrus	↑
Chau et al. (2020)	18 (15/3)	62.5 ± 3.7	Hyperlipidemia (16), hypertension (9)	15 (3/12)	71.8 ± 6.1	ROI	Global cortex, R and L cerebral, prefrontal, rostral anterior cingulate, precuneus/posterior cingulate, parietal, lateral temporal, mesial temporal, occipital, and sensorimotor cortex	↓
Huang et al. (2021)	31 (15/16)	53.4 ± 9.1	Retinopathy (31)	33 (12/21)	51.6 ± 9.8	VBA	L middle temporal gyrus, R and L supplementary motor area	↑
							R and L calcarine, and caudate	↓

*T2DM, type 2 diabetes mellitus; CBF, cerebral blood flow; ROI, region of interest; VBA, voxel-based analysis; NA, not available; R, right; L, left; n.s., no significant difference between T2DM and healthy controls; downward arrow (↓), decreased CBF in T2DM; upward arrow (↑), increased CBF in T2DM.*

**TABLE 2:** Clinical characteristics of the included studies in the meta-analysis.

References	Education (years)	Duration (year)	Onset (year)	BMI (kg/m^2^)	HbAlc (%)	MMSE
Xia et al. (2015)	9.6 ± 3.0	7.1 ± 3.5	48.9	24.4 ± 2.6	7.2 ± 1.1	29.0 ± 0.9
Jansen et al. (2016)	NA	9.8 ± 6.7	NA	29.2 ± 3.5	6.7 ± 0.4	28.6 ± 1.4
Cui et al. (2017)	10.0 ± 3.4	8.9 ± 5.0	51.6	24.4 ± 2.7	7.7 ± 1.6	28.3 ± 1.0
Dai et al. (2017)	15.4 ± 3.8	9.9 ± 7.9	55.6	29.1 ± 6.8	7.3 ± 1.25	28.6 ± 1.5
Shen et al. (2017)	9.1 ± 1.5	5.4 ± 4.9	52.2	26.0 ± 2.9	NA	NA
Zhang et al. (2019)	10.3 ± 3.7	9.2 ± 7.1	42.7	24.0 ± 3.6	NA	26.9 ± 3.9
Huang et al. (2021)	NA	NA	NA	NA	7.3 ± 1.4	NA

*T2DM, type 2 diabetes mellitus; BMI, body mass index; HbA_1c_, hemoglobin A_1c_; MMSE, Mini Mental State Examination; NA, not available.*

**TABLE 3:** Acquisition parameters and analytic methods of the included studies in the meta-analysis.

References	Acquisition parameters					Analytic methods			
	Scanner	Sequence	Labeling duration (ms)*	PLD (ms)**	Resolution (mm)	Software	FWHM (mm)	PVE correction	Threshold
Xia et al. (2015)	3T Siemens Trio	PASL	600	1000	3.4 × 3.4 × 4	SPM8	6	NA	Cluster-level FWE *p* < 0.01 corrected
Jansen et al. (2016)	3T Philips Achieva	PCASL	1,650	1,525	3 × 3 × 7	SPM8	NA	NA	FDR *p* < 0.05 corrected
Cui et al. (2017)	3T Siemens Trio	PASL	600	1,000	3.4 × 3.4 × 4	AFNI	6	GM + 0.4 × WM	AlphaSim *p* < 0.05 corrected
Dai et al. (2017)	3T GE Signa Hdxt	PCASL	1,500	1,500	1.9 × 1.9 × 4	SPM8	8	Volume of GM	Cluster-level FWE *p* < 0.05 corrected
Shen et al. (2017)	3T Siemens Skyra	PASL	Multiple TI***		3.4 × 3.4 × 4	SPM8	NA	GM + 0.4 × WM	FDR *p* < 0.05 corrected
Zhang et al. (2019)	3T GE Discovery 750	PCASL	1,525	1,525	Thickness 4	SPM8	8	Volume of brain	AlphaSim *p* < 0.01 corrected
Huang et al. (2021)	3T GE Discovery 750	PCASL	1,525	1,525	Thickness 3.5	SPM8	6	NA	Gaussian random field *p* < 0.05 corrected

*PASL, pulsed arterial spin labeling; PCASL, pseudo-continuous arterial spin labeling; PLD, post labeling delay PLD; TI, inversion time; SPM, Statistical Parametric Mapping; AFNI, Analysis of Functional NeuroImages; FWHM, full width at half maximum; PVE, partial volume effect; GM, gray matter; WM, white matter; FWE, familywise error rate; FDR, false discovery rate; NA, not available.*

*The labeling duration in PCASL is analogous to the bolus duration (TI1) in PASL.

**The PLD in PCASL is analogous to the difference between TI and TI1 in PASL.

***The Multiple TI includes 16 TIs from 480 to 4,080 ms with a step of 225 ms.

### Findings of Qualitative Review

In 3 of 6 ROI studies, researchers reported no significant regional CBF alterations in T2DM patients compared with healthy controls (Jor’dan et al., 2014; Rusinek et al., 2015; Jansen et al., 2016). The other 3 ROI studies reported significant reduction of regional CBF in T2DM patients, mainly involving frontal, temporal, and parietal lobe (Last et al., 2007; Bangen et al., 2018; Chau et al., 2020), as well as occipital lobe (Last et al., 2007; Chau et al., 2020). Another VBA study without available coordinate reported reduced CBF in insular cortex (Novak et al., 2014; Table 1).

### Findings of Meta-Analysis

**FIGURE 2:**
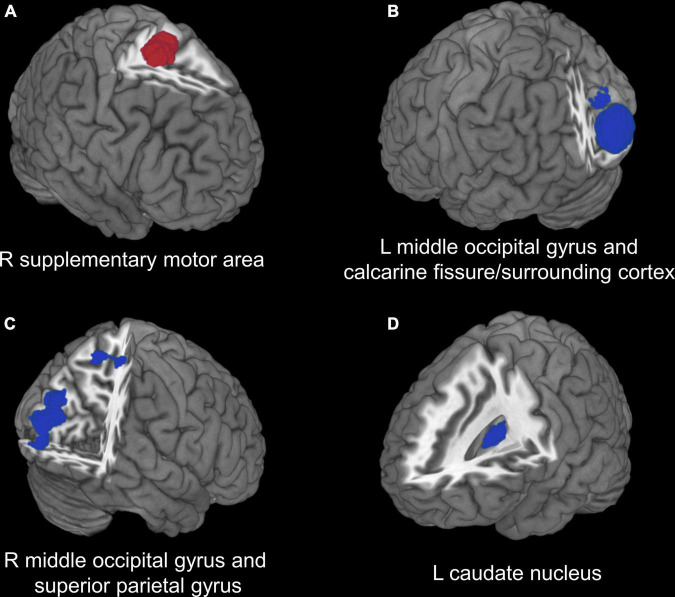
Voxel-based meta-analysis results of regions with cerebral perfusion alterations in T2DM. **(A)** Red region indicates increased CBF in patients with T2DM compared with healthy controls. **(B–D)** Blue regions indicate decreased CBF in patients with T2DM compared with healthy controls. T2DM, type 2 diabetes mellitus; CBF, cerebral blood flow; R, right; L, left.

In the voxel-based meta-analysis, 2 of 7 reports had NSUE (Jansen et al., 2016; Shen et al., 2017). Patients with T2DM showed increased regional CBF in right supplementary motor area compared with healthy controls, and decreased regional CBF in bilateral middle occipital gyrus, left caudate nucleus, right superior parietal gyrus, and left calcarine fissure/surrounding cortex (Table 4 and Figure 2).

**TABLE 4:** Differences in regional cerebral blood flow alterations between patients with T2DM and healthy controls.

	MNI coordinates	SDM-*Z* value	*p*-value	No. of voxels	Cluster breakdown (no. of voxels)
**T2DM > Control**					
R supplementary motor area	6, –12, 68	1.320	0.0002	438	R supplementary motor area (273) R superior frontal gyrus, dorsolateral (83) L supplementary motor area (40) L paracentral lobule (37) R precentral gyrus (5)
**T2DM < Control**					
L middle occipital gyrus	–18, –94, –2	–1.543	0.0004	822	L middle occipital gyrus (337) L calcarine fissure/surrounding cortex (233) L inferior occipital gyrus (128) L lingual gyrus (73) L superior occipital gyrus (46) L fusiform gyrus (3) L cuneus cortex (2)
R middle occipital gyrus	30, –90, 10	–1.380	0.0010	309	R middle occipital gyrus (194) R superior occipital gyrus (56) R inferior occipital gyrus (26) R cuneus cortex (30) L cuneus cortex (2) R calcarine fissure/surrounding cortex (1)
L caudate nucleus	–12, –2, 18	–1.365	0.0011	53	L caudate nucleus (48) L thalamus (5)
R superior parietal gyrus	16, –64, 56	–1.201	0.0023	54	R superior parietal gyrus (44) R precuneus (9) R inferior parietal gyrus (1)
L calcarine fissure/surrounding cortex	2, –86, 8	–1.227	0.0021	52	L calcarine fissure/surrounding cortex (45) L cuneus cortex (4) R calcarine fissure/surrounding cortex (3)

*T2DM, type 2 diabetes mellitus; MNI, Montreal Neurological Institute; SDM, seed-based d mapping; R, right; L, left.*

### Reliability, Heterogeneity, and Publication Bias Analyses

The jackknife analysis showed that decreased CBF in right middle occipital gyrus and right superior parietal gyrus were highly replicable and remained significant in all the combinations. The increased CBF in right supplementary motor area and the decreased CBF in left middle occipital gyrus and left calcarine fissure/surrounding cortex remained significant in 6/7 combinations. The decreased CBF in left caudate nucleus remained significant in 5/7 combinations (Supplementary Table 1).

All brain regions with CBF alterations showed low between-study heterogeneity (*I*^2^ ranged from 3.35 to 22.65%) (Supplementary Table 2). The Egger test was significant only in the right supplementary motor area (*p* = 0.001). All the brain regions with decreased CBF did not show publication bias (all *p* > 0.05) (Supplementary Table 3).

### Subgroup Meta-Analysis

The meta-analysis of PASL studies showed decreased regional CBF in right middle occipital gyrus and superior parietal gyrus. The meta-analysis of PCASL studies showed increased regional CBF in right supplementary motor area and decreased regional CBF in left middle occipital gyrus, caudate nucleus, and calcarine fissure/surrounding cortex. The meta-analysis of studies with a slice thickness 4 mm showed increased regional CBF in right supplementary motor area and decreased regional CBF in right middle occipital gyrus and superior parietal gyrus. The meta-analysis of studies using PVE correction showed increased regional CBF in right supplementary motor area and decreased regional CBF in bilateral middle occipital gyrus, right superior parietal gyrus, and left calcarine fissure/surrounding cortex (Supplementary Table 4).

### Metaregression Meta-Analysis

The metaregression analysis showed that the percentage of males, mean age, years of education, diabetic duration, onset age, BMI, HbA_1c_%, and MMSE scores were not linearly associated with regional CBF alterations in patients with T2DM.

## Discussion

To our knowledge, this is the first quantitative meta-analysis to pool the ASL studies to identify the consistent pattern of CBF alterations in T2DM. This systematic review and meta-analysis revealed that the regional CBF was significantly reduced in the patients with T2DM, mainly involving occipital and parietal lobes. These findings indicated the potential neuropathology of visual impairment and cognitive aging in T2DM (Meusel et al., 2014).

The most consistent and significant finding was that the perfusion of occipital lobe was impaired in T2DM. The middle occipital gyrus and calcarine fissure/surrounding cortex in the occipital lobe were important components of visual cortex, which were responsible for vision processing and visual memory (Tootell et al., 1998; Wandell et al., 2007). Previous studies demonstrated that the decreased perfusion in middle occipital gyrus was associated with impaired visuospatial function and visual memory (Xia et al., 2015; Cui et al., 2017). A recent meta-analysis study of functional magnetic resonance imaging (fMRI) also revealed consistent hypoactivity in the middle occipital gyrus and calcarine fissure/surrounding cortex in T2DM (Yao et al., 2021). Beside, recent studies focusing on patients with diabetic retinopathy observed decreased CBF in the bilateral calcarine fissure/surrounding cortex (Huang et al., 2021) and hypoactivity in the middle occipital gyrus (Wang et al., 2017; Qi et al., 2020). These findings indicated that the perfusion and function alterations in occipital lobe, which involving vasculopathy and neuropathy along the visual pathway (Heravian et al., 2012), might be attribute to the potential visual impairment, a common comorbidity of diabetes.

Another consistent finding was the reduced perfusion in parietal lobe in T2DM. Our quantitative meta-analysis identified decreased CBF in superior parietal gyrus. Previous neuroimaging studies also demonstrated gray matter volume loss (Roy et al., 2020) and functional dysconnectivity (Cui et al., 2016; Liu L. et al., 2017) in superior parietal gyrus in patients with T2DM. The superior parietal gyrus was involved in aspects of attention and visuospatial orientation, including the manipulation of information in working memory (Koenigs et al., 2009), which was impaired in patients with T2DM (Chen et al., 2014; Huang et al., 2016). Working memory is a fundamental cognitive process in the brain and it is crucially important for most higher-order cognitive functions (Baddeley, 2003). T2DM has been consistently associated with an increased risk of dementia and mild cognitive impairment (Reijmer et al., 2010; Beeri and Bendlin, 2020), and the structural and functional abnormalities in the brain are thought to underlie these cognitive deficits (Yao et al., 2021). Previous studies indicated that increased activation strength in parietal lobe was positively associated with memory improvement in patients with mild cognitive impairment (Belleville et al., 2011; Corriveau-Lecavalier et al., 2019). Therefore, it suggests that the decreased perfusion in superior parietal area may underlie the neuropathology of cognitive deficits in T2DM.

Our meta-analysis results also showed decreased CBF in the left caudate nucleus and increased CBF in the right supplementary motor area in T2DM, which were not commonly reported in ROI studies. Besides, it should be noted that the right supplementary motor area showed significant publication bias (Egger test *p* = 0.001). The caudate nucleus, a component of the dorsal striatum, has an important role in cognitive function and spatial working memory (Postle and D’Esposito, 2003; Grahn et al., 2008). The functional abnormalities of the caudate nucleus may also lead to motor dysfunctions (McColgan et al., 2015; Ji et al., 2018), which have been observed in patients with T2DM (Gorniak et al., 2014; Ochoa et al., 2016). Meanwhile, the supplementary motor area play a role in the direct control of movement, especially in finger movement (Shibasaki et al., 1993; Tanji and Shima, 1994), and the diabetic peripheral neuropathy may lead to sensory impairments in the motor system (Allen et al., 2016). Thus the deficits of corticostriatal circuit between the head of caudate nucleus and supplementary motor area may be the neuropathology for motor dysfunction in T2DM. The increased perfusion in supplementary motor area might suggest a compensation for the functional deficits of corticostriatal circuit in T2DM.

Although ASL has been the widely used neuroimaging approach in brain perfusion, the acquisition parameters and analytic methods varies among ASL studies, bringing potential bias. For example, quantitative assessment of perfusion with ASL is hampered by the transport time from the labeling position to the tissue, known as arterial transit time (ATT) (Alsop et al., 2015). PASL and PCASL are both labeling approaches using single PLD/inversion time (TI) but differ fundamentally in spatial extent and time of labeling and labeling delay (As shown in Table 3). Besides, one of the PASL study used multiple TI approach (Shen et al., 2017), which estimated both CBF and ATT *via* fitting data. Our subgroup meta-analysis found no overlap of regional CBF alteration between PASL and PCASL, suggesting the labeling approach might have a great impact on cerebral perfusion. As for the spatial resolution and PVE, our subgroup meta-analysis showed that the cerebral perfusion alteration in left caudate nucleus were not reproducible. One possible reason might be that the caudate nucleus was close to lateral ventricle and more likely to contain a mixture of gray matter and cerebrospinal fluid (Jezzard et al., 2018). Besides, as gray matter atrophy was observed in T2DM (Yao et al., 2021), there might be potential overestimation of decreased perfusion in regions where both perfusion and gray matter volume were reduced (Chappell et al., 2021). Future studies should attempt to conduct analysis with and without PVE correction to investigate its influence. In summary, even though some regional perfusion alterations could be affected by the heterogeneity of acquisition parameters and analytic methods, the increased CBF in right supplementary motor area and decreased regional CBF in right middle occipital gyrus and superior parietal gyrus were robust in 3 of 4 subgroup analyses.

There are several limitations in this study. First, the sample size of patients with T2DM included in some studies was relatively small. Second, near half of ASL studies in T2DM were not included in quantitative meta-analysis because of the use of ROI approach without available coordinates and corresponding effect sizes. Third, there were heterogeneity between the included studies. The confounding factors such as age, illness duration, blood glucose control, and comorbidities might affect CBF. Although we sought to identify the potential effects of some confounding factors, the results were negative, which also should be taken caution as only few data were available in the metaregression analysis. It is also difficult to avoid false-negative results even though voxel-based meta-analytical methods have good control for false-positive results (Radua et al., 2012). Fourth, although this review reveals the association between neuropathology and visual impairment and cognitive aging in T2DM, whether the vascular mechanism underlying these disorders remains inconclusive. Further research would be required to determine causation.

In conclusion, this systematic review and meta-analysis revealed consistent cerebral perfusion alterations in T2DM, characterized by decreased CBF in occipital and parietal lobes. These findings suggested the neuropathology of visual impairment and cognitive aging in T2DM.

## Data Availability Statement

The original contributions presented in the study are included in the article/Supplementary Material, further inquiries can be directed to the corresponding author/s.

## Author Contributions

JL and PZ conceived and designed the study. JL, XY, YL, and HX collected the data. JL and XY analyzed the data and drafted the manuscript. PZ revised the final manuscript. JL, JR, and PZ provided funding for the study. All authors reviewed the manuscript, contributed to the article and approved the submitted version.

## Conflict of Interest

The authors declare that the research was conducted in the absence of any commercial or financial relationships that could be construed as a potential conflict of interest.

## Publisher’s Note

All claims expressed in this article are solely those of the authors and do not necessarily represent those of their affiliated organizations, or those of the publisher, the editors and the reviewers. Any product that may be evaluated in this article, or claim that may be made by its manufacturer, is not guaranteed or endorsed by the publisher.

## Footnotes

1
http://www.sdmproject.com
