# Supplementary material for: Cerebral Blood Flow Alterations in Type 2 Diabetes Mellitus: A Systematic Review and Meta-Analysis of Arterial Spin Labeling Studies
Source: Front Aging Neurosci. 2022 Feb 16;14:847218. doi: 10.3389/fnagi.2022.847218 (PMC8888831; doi:10.3389/fnagi.2022.847218)
Supplement: Supplementary file 2 [file Table_1.DOCX]

***Supplementary Tables***

**TABLE S1.** Reliability analyses for brain regions showing cerebral blood flow alterations in patients with T2DM compared with healthy controls.

| **Jackknife analyses,**  **discarded study** | **Brain regions (MNI coordinates)** | | | | | | |
| --- | --- | --- | --- | --- | --- | --- | --- |
|  | **Increased CBF** |  | **Decreased CBF** | | | | |
|  | R supplementary motor area |  | L middle occipital gyrus | R middle occipital gyrus | L caudate nucleus | R superior parietal gyrus | L calcarine fissure/ surrounding cortex |
|  | 6,-12,68 |  | -18,-94,-2 | 30,-90,10 | -12,-2,18 | 16,-64,56 | 2,-86,8 |
| Xia et al. (2015) | Yes |  | Yes | Yes | Yes | Yes | Yes |
| Jansen et al. (2016) | Yes |  | Yes | Yes | Yes | Yes | Yes |
| Cui et al. (2017) | Yes |  | No | Yes | Yes | Yes | No |
| Dai et al. (2017) | Yes |  | Yes | Yes | No | Yes | Yes |
| Shen et al. (2017) | Yes |  | Yes | Yes | Yes | Yes | Yes |
| Zhang et al. (2019) | No |  | Yes | Yes | Yes | Yes | Yes |
| Huang et al. (2021) | Yes |  | Yes | Yes | No | Yes | Yes |
|  | 6/7 |  | 6/7 | 7/7 | 5/7 | 7/7 | 6/7 |

T2DM, type 2 diabetes mellitus; MNI, Montreal Neurological Institute; CBF, cerebral blood flow; R, right; L, left.

**TABLE S2.** Heterogeneity analysis for brain regions showing cerebral blood flow alterations in patients with T2DM compared with healthy controls.

| **Brian regions** | **MNI coordinates** | ***I^2^* (%)** |
| --- | --- | --- |
| **T2DM > Control** |  |  |
| R supplementary motor area | 6,-12,68 | 17.59 |
| **T2DM < Control** |  |  |
| L middle occipital gyrus | -18,-94,-2 | 22.65 |
| R middle occipital gyrus | 30,-90,10 | 5.49 |
| L caudate nucleus | -12,-2,18 | 9.28 |
| R superior parietal gyrus | 16,-64,56 | 3.37 |
| L calcarine fissure/surrounding cortex | 2,-86,8 | 3.35 |

T2DM, type 2 diabetes mellitus; MNI, Montreal Neurological Institute; R, right; L, left.

**TABLE S3.** Funnel plots and Egger test for brain regions showing cerebral blood flow alterations in patients with T2DM compared with healthy controls.

| **Brian regions** | **MNI coordinates** | **Funnel plots** | **Egger test** |
| --- | --- | --- | --- |
| **T2DM > Control** |  |  |  |
| R supplementary motor area | 6,-12,68 | 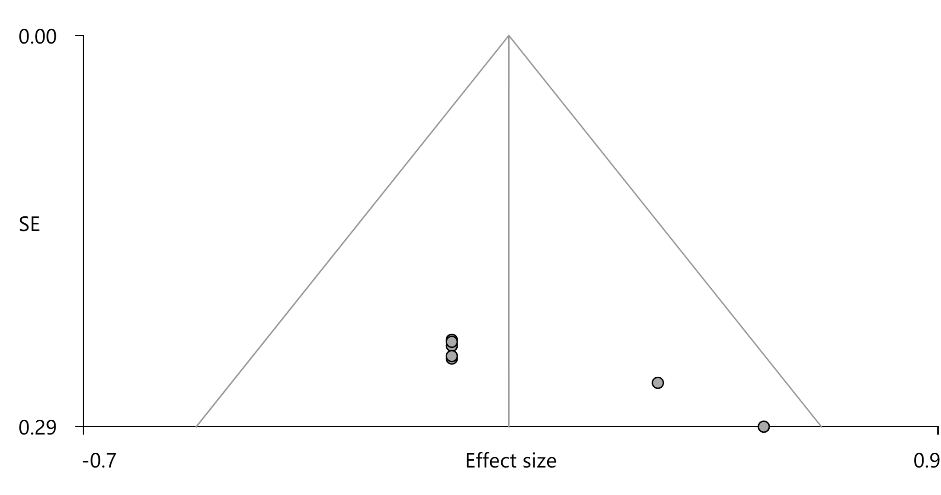 | *t* = 6.65, *p* = 0.001 |
| **T2DM < Control** |  |  |  |
| L middle occipital gyrus | -18,-94,-2 | 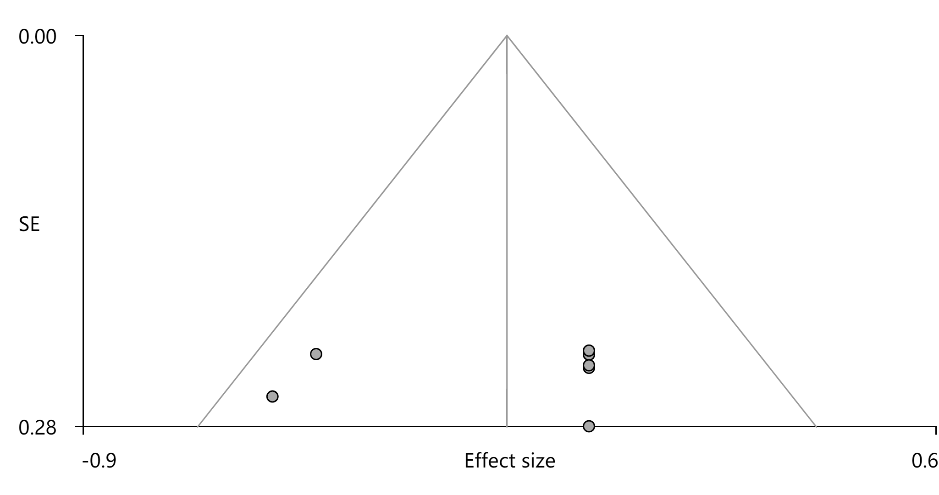 | *t* = -0.24, *p* = 0.82 |
| R middle occipital gyrus | 30,-90,10 | 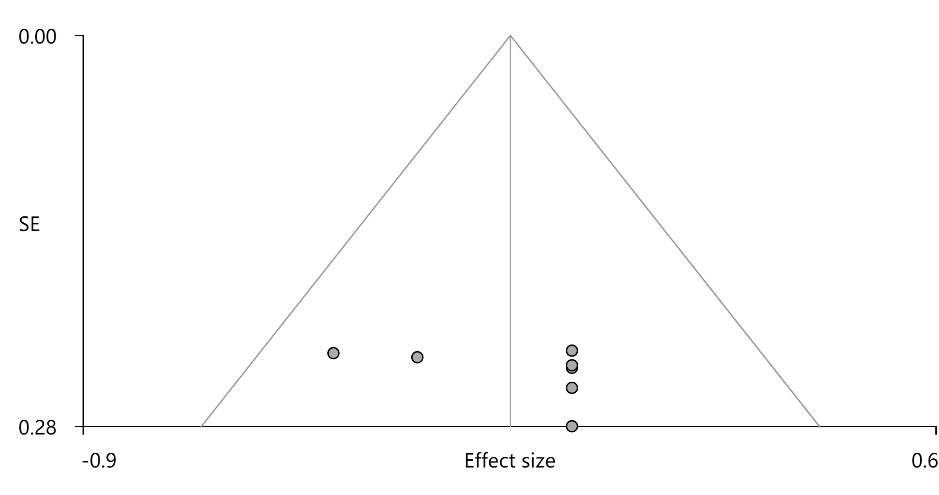 | *t* = 1.05, *p* = 0.34 |
| L caudate nucleus | -12,-2,18 | 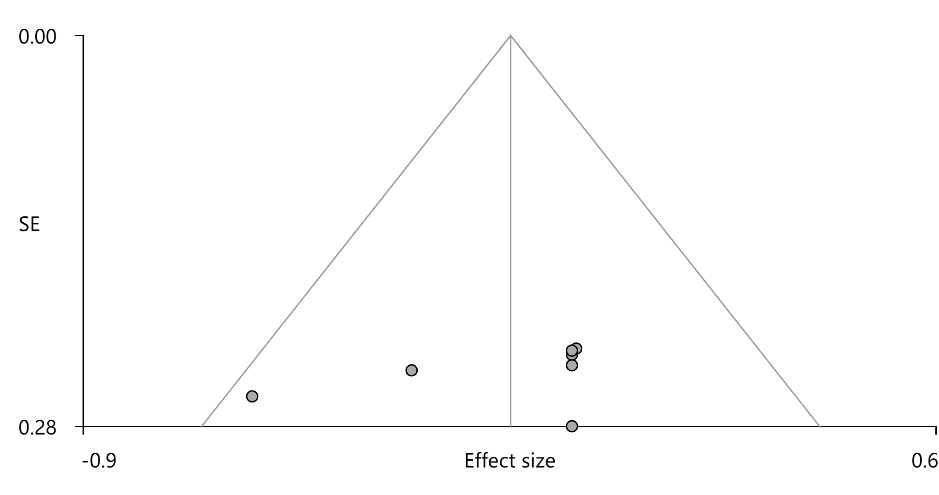 | *t* = -0.98, *p* = 0.37 |
| R superior parietal gyrus | 16,-64,56 | 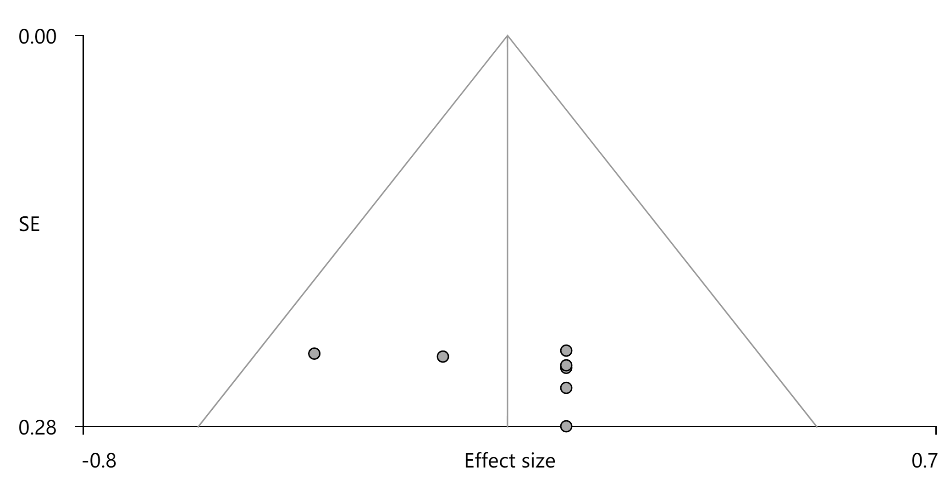 | *t* = 1.01, *p* = 0.36 |
| L calcarine fissure/surrounding cortex | 2,-86,8 | 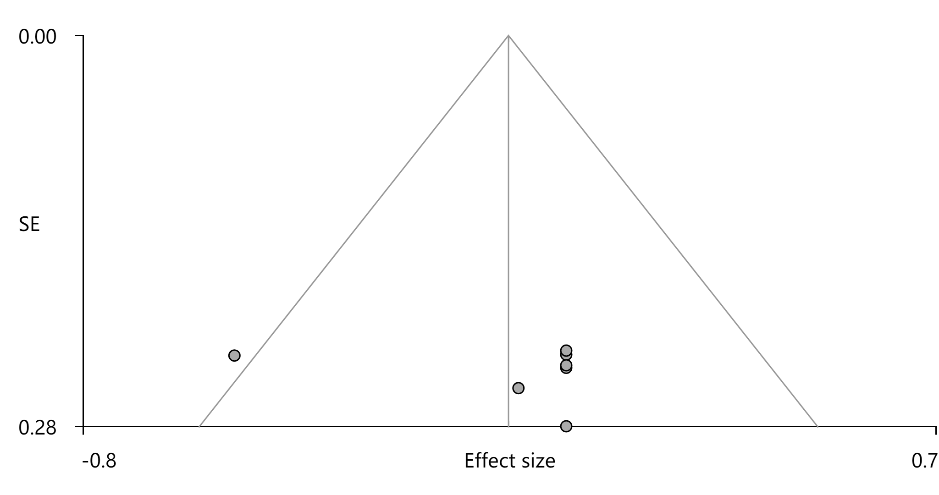 | *t* = 0.52, *p* = 0.63 |

T2DM, type 2 diabetes mellitus; MNI, Montreal Neurological Institute; R, right; L, left.

**TABLE S4.** Subgroup analyses for brain regions showing cerebral blood flow alterations in patients with T2DM compared with healthy controls.

| **Subgroup analyses** | **Brain regions (MNI coordinates)** | | | | | | |
| --- | --- | --- | --- | --- | --- | --- | --- |
|  | **Increased CBF** |  | **Decreased CBF** | | | | |
|  | R supplementary motor area |  | L middle occipital gyrus | R middle occipital gyrus | L caudate nucleus | R superior parietal gyrus | L calcarine fissure/ surrounding cortex |
|  | 6,-12,68 |  | -18,-94,-2 | 30,-90,10 | -12,-2,18 | 16,-64,56 | 2,-86,8 |
| PASL (n = 3) | No |  | No | Yes | No | Yes | No |
| PCASL (n = 4) | Yes |  | Yes | No | Yes | No | Yes |
| Slice thickness = 4 mm (n = 5) | Yes |  | No | Yes | No | Yes | No |
| PVE correction (n = 4) | Yes |  | Yes | Yes | No | Yes | Yes |

T2DM, type 2 diabetes mellitus; MNI, Montreal Neurological Institute; CBF, cerebral blood flow; R, right; L, left; PASL, pulsed arterial spin labeling; PCASL, pseudo-continuous arterial spin labeling; PVE, partial volume effect.
